# Dietary Patterns in Asia: Current Evidence and Future Directions

**DOI:** 10.1016/j.advnut.2024.100250

**Published:** 2024-06-20

**Authors:** Lukas Schwingshackl, Georg Hoffmann

**Affiliations:** 1 Institute for Evidence in Medicine, Faculty of Medicine and Medical Center, University of Freiburg, Freiburg, Germany; 2 Department of Nutritional Sciences, University of Vienna, Vienna, Austria

More than 20 y ago, dietary pattern analysis has emerged as a remarkable approach to examine the relationship between diet and risk of noncommunicable diseases. Instead of focusing on individual nutrients or foods, dietary pattern analysis examines the health impact of overall diet [1]. With respect to assessment of diet quality, this approach has distinct advantages when compared with looking at nutrients alone (among other things, they come closer to the “nutritional reality” of the individuum). Dietary patterns are mainly evaluated by the following: *1*) an index-based or a priori approach, measuring conformity to dietary guidelines using predefined dietary indices intended to capture specific dietary patterns from epidemiologic research findings, such as the Alternate Healthy Eating Index (AHEI); or *2*) a data-driven (exploratory) or a posteriori approach, which uses population-based dietary intake data to derive possible consumption patterns (e.g., prudent dietary pattern) via statistical methods (principal component analysis and factor analysis) [2]. Both approaches and the corresponding statistical methods have their own specific strengths and limitations. Since then, a plethora of evidence on the association between adherence to diverse dietary patterns and risk of noncommunicable diseases, such as cardiovascular diseases (CVD), have been published [2,3].

As highlighted in the article by Lim et al. [4], CVD is a leading cause of death worldwide, with a high proportion of premature CVD deaths occurring in Asia, and optimal diet plays a major role in CVD prevention. It is well known that Japan has one of the longest average life expectancy worldwide and low prevalence rates of ischemic heart disease and obesity. The Japanese dietary pattern is characterized by low intake of red meat, high intakes of fish, plant foods (especially soybeans), and nonsugar-sweetened beverages [5]. To shed light on the influence of dietary patterns in Asian populations, Lim et al. [4] collected the available evidence for several high-quality and low-quality dietary patterns including Asian and non-Asian dietary indices, and data-driven dietary patterns. Overall, the authors enclosed 41 relevant studies and showed that several high-quality dietary patterns (Asian, non-Asian, and data-driven patterns) were consistently related to lower CVD risk (16%–22%). On the contrary, plant-based indices, low-quality, animal-based, and diet diversity indices dietary patterns were not associated with CVD risk. The authors note that given the evidence that associations between current Asian-derived diet quality indices (relative risk: 0.84; 95% CI: 0.79, 0.90) and CVD risk were weaker than those between established non-Asian diet quality indices (relative risk: 0.78; 95% CI: 0.69, 0.88) such as AHEI, there may be a need for current dietary guidelines in Asian countries and Asian diet quality indices to be optimized to reflect up-to-date dietary recommendations for cardiovascular health.

However, when evaluating the impact of dietary patterns on people’s health, it is necessary to quantify a quality. Thus, dietary patterns are quantified using scoring systems that are not always clear and consistent. For example, there is >1 scoring system proposed to assess the adherence to a Mediterranean diet [6], which are not congruent in every respect. The difficulty of clearly defining dietary patterns and distinguishing them from other patterns also arises in the work of Lim et al. [4], quite transparent in Supplemental Table 4. The authors address some of the problems they had in the discussion: food groups such as fish, seafood, or poultry are not only assigned to a high-quality pattern but also part of animal-based dietary patterns classified as low-quality. Another point of criticism could be the separate use of a plant-based dietary pattern, which is defined by the authors as “comprising studies that adopted a scoring algorithm to measure consumption of healthy plant foods.” This sounds a bit artificial, after all, the focus on plant-based food groups is a characteristic trait of most dietary patterns exhibiting beneficial effects on human health. The fact that this subgroup had no benefit in this study should not lead to the conclusion that a plant-based diet has no such protective effects.

This raises the question of how dealing with dietary patterns can be improved, for example, with respect to either clearer differentiation or comparability. Lim et al. [4] suggested to adapt established patterns (e.g., AHEI) to geographical features, that is, nutritional habits in other regions. In the past, such an attempt was made by Anand et al. [7] for the Mediterranean diet. Lim et al. [4] recommend focusing on the intake of soy, fermented soy products, or lentils instead of tree nuts or legumes for the Asian region. Given the potential mechanisms of action, this only makes sense if these food groups can be exchanged in terms of not only eating habits but also the effects of their ingredients. Such a breakdown of a complex pattern into the effect of certain ingredients has been described in a review of the Mediterranean diet [8]. In addition, in several studies on tree nuts, the beneficial effect of a regular consumption was explained via their dominant constituents [9]. Comparable positive effects have been described for soy [10]. Owing to the changes in the composition of bioactive metabolites, consumption of fermented soy products could even provide a stronger benefit [11]. Therefore, adapting evidence-based patterns to regional dietary habits represents a promising tool in the formulation of nutritional recommendations.

Finally, although the call for optimization of dietary guidelines and diet quality indices for Asian countries as expressed by Lim et al. [4] sounds reasonable, the underlying results need to be interpreted with caution. The authors did not conduct a formal statistical test for subgroup differences, comparing the relation of non-Asian diet with that of non-Asian quality indices on CVD risk. Such a test would clearly indicate that there are no statistically significant differences between the 2 indices, which is also shown by the overlap of the CIs of the 2 summary estimates. Moreover, in order to prove the trustworthiness of a subgroup effect, the usage of the ICEMAN instrument is recommended [12]. Despite this limitation, the systematic review by Lim et al. [4] is well conducted as the authors used state-of-the-art methodologic approaches including a prespecified analysis plan, risk of bias assessment with the ROBINS-E tool, and certainty of evidence assessment using the Grading of Recommendations, Assessment, Development, and Evaluations (GRADE) approach.

## Author contributions

The authors’ responsibilities were as follows – LS, GH: drafted the manuscript; and both authors read and approved the final manuscript.

## Conflict of interest

LS is an Associate Editor of *Advances in Nutrition* and played no role in the Journal’s evaluation of the manuscript. LS is a member of the Grading of Recommendations, Assessment, Development and Evaluations (GRADE) working group. GH has no conflict of interest.
